# Assessment of heavy metals among auto workers in metropolitan city: a case study

**DOI:** 10.3389/fpubh.2023.1277182

**Published:** 2023-11-07

**Authors:** Kaleem Khan, Shahzada Amani Room, Aziz-Ur-Rahim Bacha, Iqra Nabi, Shabir Ahmad, Muhammad Younas, Zahid Ullah, Akhtar Iqbal, Abdulwahed Fahad Alrefaei, Mikhlid H. Almutairi, Jung-Wei Chang, Kai Hsien Chi

**Affiliations:** ^1^Institute of Environmental and Occupational Health Sciences, School of Medicine, National Yang Ming Chiao Tung University, Taipei, Taiwan; ^2^Department of Environmental Science, Faculty of Basic and Applied Sciences, International Islamic University, Islamabad, Pakistan; ^3^State Key Laboratory of Urban Water Resource and Environment, Shenzhen Key Laboratory of Organic Pollution Prevention and Control, School of Civil and Environmental Engineering, Harbin Institute of Technology Shenzhen, Shenzhen, China; ^4^College of Resources and Environment, Huazhong Agricultural University, Wuhan, China; ^5^State Key Laboratory of Biogeology and Environmental Geology, School of Environmental Studies, China University of Geosciences, Wuhan, China; ^6^Department of Environmental Sciences, COMSATS University Islamabad, Abbottabad Campus, Abbottabad, Pakistan; ^7^Department of Zoology, College of Science, King Saud University, Riyadh, Saudi Arabia

**Keywords:** heavy metals, autoworkers, mechanics, spray painters, battery recyclers

## Abstract

In recent decades, heavy metals (HMs) have emerged as a global health concern. Unfortunately, in Pakistan, there is a general lack of awareness regarding the potential health risks associated with HMs pollution among automobile workers. Herein, we investigated the concentration of heavy metals such as lead (Pb), cadmium (Cd), and chromium (Cr) among automobile workers who were occupationally exposed in Mingora City, Khyber Pakhtunkhwa, Pakistan. Three different automobile groups, i.e., battery recyclers, spray painters, and mechanics were studied in detail. A total of 40 blood samples were collected from automobile workers groups while 10 blood samples were collected as control individuals from different locations in the study area. We investigated heavy metals concentration with a standard method using an atomic absorption spectrometer AAS (PerkinElmer Analyst 700, United States). Based on our findings, the battery recycling group displayed the most elevated Pb levels (5.45 ± 2.11 μg/dL), exceeding those of both the spray painters’ group (5.12 ± 1.98 μg/dL) and the mechanics’ group (3.79 ± 2.21 μg/dL). This can be attributed to their higher exposure to Pb pollution resulting from the deterioration, dismantling, grinding, or crushing of old batteries. In the context of chromium (Cr) exposure, a similar trend was observed among the battery recycling group, as well as the spray painters and mechanics groups. However, in the case of cadmium (Cd), the mechanics’ group exhibited the highest level of exposure (4.45 ± 0.65 μg/dL), surpassing the battery recycling group (1.17 ± 0.45 μg/dL) and the spray painters’ group (1.35 ± 0.69 μg/dL), which was attributed to their greater exposure to welding fumes and other activities in their workplace. We believe that our findings will encourage regulatory measures to improve the health of automobile workers. However, further work is needed to determine various health-related issues associated with heavy metal exposure among automobile workers.

## Graphical abstract

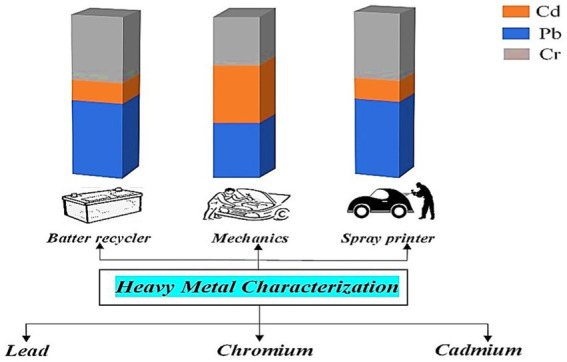

## 1. Introduction

Metals are natural constituents having a high electrical conductivity that occurs in the biological system. They are present everywhere throughout the earth and may also accumulate in living organisms as well. Among 35 natural obtainable metals, 23 metals (lead, cadmium, chromium, mercury, copper, cobalt, arsenic, nickel, platinum, silver, manganese, antimony, cerium, gallium, uranium, iron, tellurium, bismuth, tin, thallium, gold, zinc, and vanadium) have high obvious density with an atomic weight greater than 40.04 are known as heavy metals (1, 2). These metals are not only known for their high density and conductivity but they also have an adverse effect on the biological system (3). In the human body, these metals are present in body tissues, nucleic acids, and proteins which leads to disturbance in working abilities (4). Besides, when these metals are used in industries, a portion of these components are discharged into the air or water bodies as effluents and may indirectly affect humans and other living organisms. Heavy metals (HMs) pollution is a global issue while the associated ecological and health risks are not yet understood due to its vast distribution. Moreover, unspecialized manufacturing and industrial activities leads to an increased discharge into different environmental media (5).

All workplaces have some specific levels of work-related dangers, and each workplace condition is remarkable in nature. Synthetic mixtures are discharged at a workplace from various operations such as residue or splash. It may get into the body either through dermal contact, inhalation, or ingestion (6). Workers commonly eat, drink, and smoke at the workplace due to a lack of knowledge and awareness, and such practices increase their susceptibility to toxic substances (7, 8).

Generally, automobile workshops consist of different groups such as mechanics, spray painters, battery recyclers, and radiator workers for specific tasks. However, these certain activities are the major sources of toxic pollution due to the irresponsible behavior of the occupational workforce. Several studies have suggested that autoworkers are commonly exposed group to toxic metal pollution (9, 10). While others reported that spray painters are at higher risk of cadmium (Cd), chromium (Cr), and lead (Pb) toxicity in automobile paint workshops (11). Conversely, Cd, Cr, and Pb are the most toxic metals that are commonly present in paints (12). Workers in automobile workshops are described as being affected by toxic chemicals like lead fumes, carbon dioxide fumes, chromium, and benzene fumes. It is also reported that lead, chromium, and cadmium are components of spare parts used in the vehicle manufacturing industry, which might influence the levels of these metals in the blood serum of professional workers having regular interaction with them (13, 14). Autoworkers’ exposure to these fumes may occur through inhalation during melting processes and absorbed or ingested through the skin when a panel beater regularly uses the metals for repair (13). Although, long-term exposure to Cd may cause several health problems such as dysfunction of the cardiovascular, immune, nervous, respiratory, and endocrine systems. In addition, it may also cause lung cancer in workers (15, 16). Whereas these chemical substances are released in the workplace due to the usage of spray and dust which enters the human body through inhalation, ingestion, as well as dermal contact as a result heavy metals, are a serious concern (17, 18).

The present study was conducted in Mingora city district Swat where automobile workshops are situated in various locations and the reason that these sites are most familiar for different vehicular activities. This study reveals that some automobile workshops were small having 4–5 workers, while others were large having 8–10 working groups. Unfortunately, these automobile workers have no knowledge and awareness about the toxic effects of HMs, nor do they use any protective measures, as a result, they pay slight consideration to protect themselves from the possible impacts due to ingestion and inhalation of these toxic substances. Herein, the evaluations of selected HMs concentrations of Pb, Cd, and Cr were done in exposed autoworkers and a comparison was done with a control group (non-workers).

## 2. Materials and methods

### 2.1. Study site

**Figure 1:**
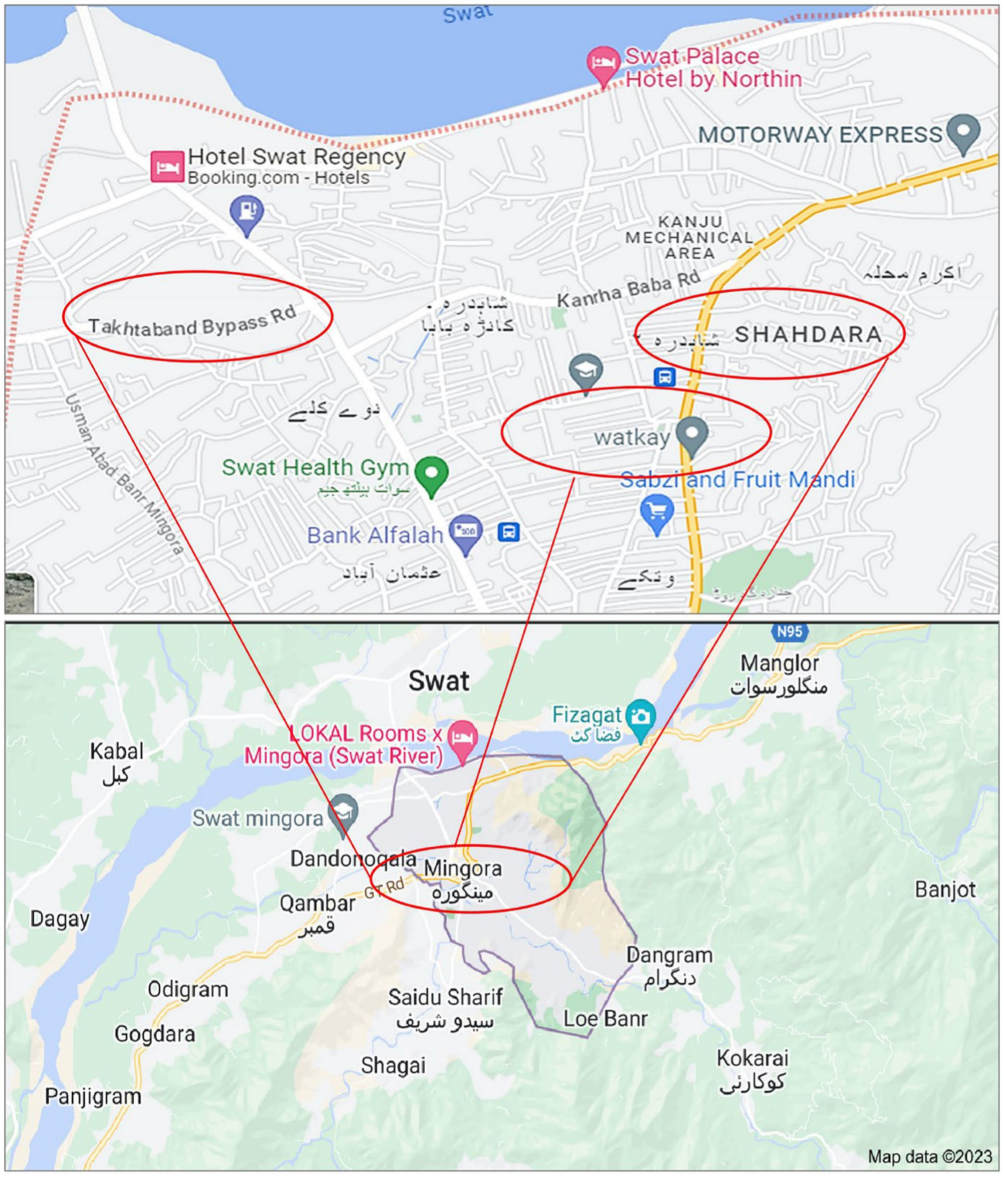
Study map of samples collection in district Swat, Khyber Pakhtunkhwa province in Pakistan.

The study area is located in the district Swat of Khyber Pakhtunkhwa (KPK) province, Pakistan, and lies between 34° 46′ 25.1292″ north and 72° 21′ 35.6436″ east. Mingora is the third-largest city in KPK with a high population ratio. This study focused on occupationally exposed autoworkers of Mingora city, district Swat. A total number of 40 autoworkers including mechanics, spray painters, and battery repairing, and 10 non-workers (control group) have participated in this study from different locations including Takhtaband Bypass Rd., Shahdara, and Watkay site, respectively, as shown in Figure 1. The workers’ groups were engaged in different activities in workshops, whereas the controls were not directly exposed to such kind of contamination.

All these workers had been working in workshops for 4 to 5 years or more than 10 years, while the workers who had joined workshops for less than 1 year were excluded from this survey. Furthermore, all the workers were interviewed regarding workplace safety precautions. However, it was observed that all the exposed autoworkers had a lack of awareness regarding workplace safety measures.

### 2.2. Samples collection

Several workshops were visited, and the purpose of the study was discussed with the workers and non-worker groups. The samples were collected from different automobile workshops including mechanics, battery recyclers, and spray painters. Each 3 mL blood sample was collected in heparinized tubes using the standard method. Blood was taken with the help of a trained nurse from both autoworkers and non-worker groups. After collection, blood samples were directly subjected to the laboratory for further experimental work.

### 2.3. Samples preparation and analysis

The sample preparation and analysis were carried out according to the previous method of Ishola et al. (19) with a slight modification. Each blood sample tube was centrifuged at 4,000 rpm for 10 min for the separation of blood supernatant serum. The supernatant serum was kept in a separate tube. The prepared samples were then stored at −200°C for at least 10–12 days before analysis. The levels of lead, cadmium, and chromium were then analyzed by using atomic absorption Spectrometer AAS (PerkinElmer A Analyst 700, United States) respectively. The mean value for each sample was calculated using three measurement readings, and the error estimate was calculated via the standard deviation of the results.

### 2.4. Statistical analysis

Data were analyzed through the Statistic 9 software. One-way ANOVA was performed considering the (*p* < 0.05). Furthermore, graphs were made by using the graph pad prism version (5.01).

## 3. Results and discussion

**Figure 2:**
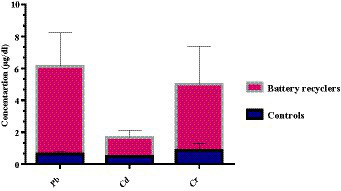
Heavy metals concentrations (μg/dL) in battery recyclers automobile group.

The average concentration of HMs found in the blood serum collected from automobile workers, including mechanics, battery recyclers, spray painters, and the control group. As a result, the battery recyclers group exhibited a higher Pb value (5.45 ± 2.11 μg/dL), surpassing both the spray painters’ group (5.12 ± 1.98 μg/dL) and mechanics group (3.79 ± 2.21 μg/dL). The mean values of Pb, Cd, and Cr were recorded higher in the mechanic, battery recyclers, and spray painters’ group whereas their mean values were recorded lower in the control group as presented in Table 1 and Figure 2. The result in Figure 2 shows that the mean value of Pb in the battery recyclers group was higher in comparison with Cr and Cd while the concentration of these HMs follows the order Pb > Cr > Cd.

**Table 1:** Mean concentrations of metals (μg/dL) in mechanics, battery recyclers, and spray painters group compared to the controls.

Parameters	Controls group	Mechanics group	Battery recyclers group	Spray painters’ group	*p*-value
Lead	0.64 ± 0.15	3.79 ± 2.21	5.45 ± 2.11	5.12 ± 1.98	0.001
Cadmium	0.49 ± 0.012	4.45 ± 0.65	1.17 ± 0.45	1.35 ± 0.69	0.001
Chromium	0.87 ± 0.41	4.38 ± 2.76	5.13 ± 2.37	4.72 ± 2.27	0.001

A high concentration of Pb was observed in battery workers as compared to automobile workers in Pakistan Ahmad et al. (20). Meanwhile, a similar phenomenon of Pb concentration was noticed in battery workers in Colombia Restrepo et al. (21). Our findings are consistent with the reported literature. Pb is commonly utilized in various applications, including lead-acid batteries, projectiles, and ammunition (22). The differences in these values were attributed to the higher exposure of battery recyclers to Pb, which includes activities such as deterioration, dismantling, grinding, or crushing of old batteries, respectively. As reported by Gottesfeld and Pokhrel (23), approximately (50%) of the lead production in automotive workshops is attributed to the recycling of lead batteries. In Pakistan, it has been estimated that approximately (95%) of lead-acid batteries are produced by reutilizing old batteries. Moreover, the elevated levels of Pb in battery recyclers can be attributed to inadequate personal hygiene practices, as well as the consumption of contaminated food and water at their workplace (24). Our findings for Pb concentration are in good agreement with the reported literature (9, 25, 26).

**Figure 3:**
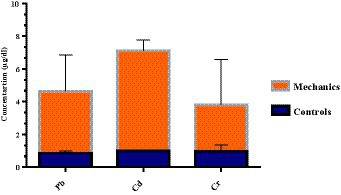
Heavy metals concentrations (μg/dL) in mechanics automobile group.

Moreover, in Table 1, it was observed that the mechanic’s group had the maximum level of Cd (4.45 ± 0.65 μg/dL), while the minimal level was documented in the battery recyclers group (1.17 ± 0.45 μg/dL). On the other hand, the spray painters’ group had an intermediate level of Cd (1.35 ± 0.69 μg/dL). The concentration of these HMs in the mechanic’s group follows the order Cd > Pb > Cr as shown in Figure 3. Cd is primarily linked with exposure to welding fumes in auto workshops (27). As well as, Cd can also accumulate within the body through various means such as inhalation, consumption of contaminated food, and improper washing practices in the workplace (28). According to Goyal et al. (29), auto workers who were exposed to welding fumes through inhalation within the workplace exhibited higher levels of cadmium in their bodies. Similarly, Ishola et al. (19) have also documented similar findings concerning the levels of Cd in the blood of automobile workers in Benin City, Nigeria. Furthermore, spray painters use high compression to apply paints onto vehicle body parts, causing them to become aerosolized and dispersed into the surrounding environment (30). In addition, during our study, we observed that the spray painters did not use proper respiratory protection such as masks or protective clothing. As a result, they were directly exposed to these toxicants through inhalation, ingestion, and dermal contact while performing their work (31). reported that individuals who work with spray printers face potential exposure to Cr through both inhalation and ingestion.

**Figure 4:**
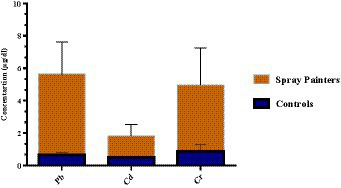
Heavy metals concentrations (μg/dL) in spray painters automobile group.

In the present study, a significant variation was observed in the level of Cr among different groups. The battery recyclers group exhibited a higher mean value of Cr (5.13 ± 2.37 μg/dL), whereas the mechanic’s group had a lower mean value (4.38 ± 2.76 μg/dL), however, the spray painters group showed an intermediate level of Cr (4.72 ± 2.27 μg/dL) when compared to a control group (0.87 ± 0.41 μg/dL). However, the concentration of these HMs in the spray painters group follows the order Pb > Cr > Cd as shown in Figure 4. Metal-coated welding electrodes, fumes, and lubricants are also the main sources of chromium pollution. Ahmad et al. (32) stated that exposure to Cr typically occurs during various activities such as coating, painting, welding, cutting, and metal treatment. Autoworkers are usually exposed to Cr pollution through (inhalation, ingestion of contaminated food, etc.) and similar results have been reported in the literature (33, 34).

## 4. Conclusion

In the present work, it is concluded that the levels of lead and chromium in the battery recycling group were significantly higher when compared to both the mechanics and spray printer groups due to their higher exposure to different activities in their work. Moreover, in the case of Cd, the Mechanics groups have the maximum concentrations compared to other groups. Nonetheless, the concentration of all HMs among the autoworker’s groups was greater in comparison to control individuals. In the present study, we noticed that workers did not utilize any personal protective equipment and had limited awareness regarding the toxic effects of heavy metals released in their workplaces. However, here are some measures including regular cleanup, safe handling and storage, proper education and awareness, training, regular assessment and improvement, regular health checking, and compliance with regulations might be helpful for workers to reduce potential exposure to heavy metals and other toxic chemicals in workshops.

## Data availability statement

The original contributions presented in the study are included in the article/supplementary material, further inquiries can be directed to the corresponding authors.

## Ethics statement

The studies involving humans were approved by the Ethics Committee of the International Islamic University Islamabad (IIUI). Consent was obtained from all the individual participants included in the study. The studies were conducted in accordance with the local legislation and institutional requirements. Written informed consent for participation in this study was provided by the participants’ legal guardians/next of kin.

## Author contributions

KK: Formal analysis, Investigation, Writing – original draft. SR: Writing – review & editing. A-U-RB: Supervision, Writing – original draft, Writing – review & editing. IN: Writing – review & editing. SA: Writing – review & editing. MY: Writing – review & editing. ZU: Supervision, Writing – review & editing. AI: Writing – review & editing. AA: Funding acquisition, Writing – review & editing. MA: Funding acquisition, Writing – review & editing. J-WC: Writing – review & editing. KC: Writing – review & editing.
